# Increased Risk of Age-Related Macular Degeneration with Chronic Hepatitis C Virus Infection: A Nationwide Population-Based Propensity Score-Matched Cohort Study in Taiwan

**DOI:** 10.3390/v13050790

**Published:** 2021-04-28

**Authors:** Chih-Ching Yeh, Meei-Maan Wu, Chia-Min Wu, Fung-Chang Sung, Chih-Hsin Muo, Arlene Te, Fu-Hsiung Su

**Affiliations:** 1 School of Public Health, College of Public Health, Taipei Medical University, Taipei 110, Taiwan; ccyeh@tmu.edu.tw (C.-C.Y.); mmwu@tmu.edu.tw (M.-M.W.); windex034@gmail.com (C.-M.W.); 2 Department of Public Health, College of Public Health, China Medical University, Taichung 404, Taiwan; 3 Cancer Center, Wan Fang Hospital, Taipei Medical University, Taipei 116, Taiwan; 4 Master Program in Applied Molecular Epidemiology, College of Public Health, Taipei Medical University, Taipei 110, Taiwan; 5 Department of Public Health, School of Medicine, College of Medicine, Taipei Medical University, Taipei 110, Taiwan; 6 Department of Ophthalmology, Shuang Ho Hospital, Taipei Medical University, New Taipei City 235, Taiwan; 7 Management Office for Health Data, China Medical University Hospital, Taichung 404, Taiwan; fcsung1008@yahoo.com (F.-C.S.); b8507006@gmail.com (C.-H.M.); 8 Department of Health Services Administration, China Medical University, Taichung 406, Taiwan; 9 Department of Food Nutrition and Health Biotechnology, Asia University, Taichung 413, Taiwan; 10 Department of Family Medicine, Cardinal Tien Hospital, Fu Jen Catholic University, New Taipei City 231, Taiwan; srarlene@yahoo.com; 11 School of Medicine, College of Medicine, Fu Jen Catholic University, New Taipei City 242, Taiwan

**Keywords:** hepatitis C virus, aged-related macular degeneration, propensity score matching, insurance data, retrospective cohort study

## Abstract

Studies evaluating the association between age-related macular degeneration (AMD) risk and HCV infection are scant. In this population-based cohort study, 13,300 patients newly diagnosed as having HCV (HCV cohort) and 26,600 propensity score-matched patients without HCV (non-HCV cohort) were identified from the Taiwan National Health Insurance Research Database between 2000 and 2013. Furthermore, 1,983 patients with HCV who received pegylated interferon and ribavirin treatment (HCV-treated cohort) and propensity score-matched patients with HCV (matched at a ratio of 1:2) who did not receive this treatment (HCV-untreated cohort) were selected from the HCV cohort. Cox proportional hazards regression models were used to calculate hazard ratios (HRs) and 95% confidence intervals (CIs) associated with the risk of AMD in the HCV and non-HCV cohorts. The adjusted HR (aHR) for AMD in the HCV cohort was 1.22 (95% CI = 1.09–1.35). This significant association was observed only for nonexudative AMD (aHR = 1.22, 95% CI = 1.09–1.37). Compared with the HCV-untreated cohort, the HCV-treated cohort showed no significant association with any type of AMD (aHR = 1.07, 95% CI = 0.81–1.43). Age and sex did not modify AMD development after the exposure and treatment of chronic HCV infection. Our findings revealed that patients with chronic HCV infection had an increased risk of AMD.

## 1. Introduction

Age-related macular degeneration (AMD) is a disease that affects the macular region of the retina and is the leading cause of vision loss in the older population in Western countries [1]. Clinically, it is categorized as early-stage and late-stage [2]. The clinical signs of early-stage AMD include the development of drusen and abnormalities of the retinal pigment epithelium. Late-stage AMD can be neovascular (also known as exudative) or non-neovascular (known as atrophic or non-exudative). Late AMD can lead to the loss of central visual acuity and, consequently, result in severe and permanent visual impairment. Hence, AMD has tremendous effects on quality of life and functional independence [2]. The global prevalence of any, early, and late AMDs was estimated to be 8.7%, 8.0%, and 0.4%, between the age of 45 and 85, respectively. In 2015, AMD was ranked as the fourth and third most common causes of blindness (5.8%) and moderate-to-severe vision impairment (3.9%) globally, respectively [3]. A study in Taiwan found the prevalence of AMD in a study is comparable to that reported in previous international studies [4].

AMD is a multifactorial disorder. In addition to age, sex, and genetics, which have been identified as unmodifiable risk factors for AMD, many socioeconomic, lifestyle (e.g., cigarette smoking), environmental, and cardiovascular factors have been indicated as potential risk factors [2]. Infectious agents, such as cytomegalovirus [5], hepatitis B virus (HBV) [6,7], and human immunodeficiency virus [8], have been suggested to be related to AMD development. The administration of high-dose zinc and antioxidant vitamin supplements may slow down the progression from early- to late-stage AMD [2]. Although intravitreal anti-vascular endothelial growth factor therapy is a highly feasible treatment modality for treating neovascular AMD, no proved therapy is available for non-neovascular AMD yet [2]. Identification and prevention of modifiable risk factors of AMD are hence important strategies to alleviate vision loss.

Hepatitis C virus (HCV) is a type of a human hepatotropic virus with considerable effects globally because HCV infection can progress to liver cirrhosis and eventually to hepatocellular carcinoma [9]. In 2005, approximately 185 million people worldwide were diagnosed as having HCV infection. The prevalence of anti-HCV antibodies has been estimated to range from 1.7% to 2.8% in adults worldwide [9,10]. Taiwan is one of the areas with a high prevalence of HCV infection, with a rate of 4.4% in people aged ≥20 years. The prevalence of HCV infection increases with age [11].

Because HCV is lymphotropic and hepatotropic in nature, it not only causes hepatic diseases but also is commonly associated with many extrahepatic manifestations. The well-known extrahepatic complications of HCV infection are cryoglobulinemia, glomerulonephritis, Sjögren’s syndrome, high autoantibody titers, idiopathic thrombocytopenic purpura, necrotizing cutaneous vasculitis, lichen planus, Mooren’s corneal ulcer, and porphyria cutanea tarda [12]. HCV-related extrahepatic manifestations are considered to be most likely involved in autoimmune mechanisms. This evidence is supported by the occurrence of immune features and the development of immune-complex deposit diseases that subsequently cause local and systemic complications [12].

Many studies have reported the detection of HCV and its antigen in the tears and aqueous humor of seropositive patients [13,14,15,16,17,18]. In addition, HCV infection has been indicated to be associated with many ocular pathophysiologies such as acute vision loss [19], retinopathy [20,21], retinal pigment epithelitis [22], uveitis [23], cataract [24], and dry eye disease [25,26]. Recently, dry eye disease [25] and ischemic retinopathy caused by either HCV-induced vasculitis [20] or interferon treatment [25,26] have been reported to be strongly associated with the ocular manifestations of HCV infection. The pathogenesis of ocular changes may be the direct action of viral and immunological reactions to certain viral antigens and even immune complexes [27]. However, evidence suggesting an association of between HCV infection and AMD is limited.

Both AMD and HCV infections are common in Taiwan. Therefore, the present study evaluated whether patients with chronic HCV infection have an increased risk of AMD and whether the risk of AMD is associated with interferon treatment for HCV infection by using the nationwide population-based insurance claims data of a Taiwanese population aged ≥18 years.

## 2. Materials and Methods

### 2.1. Data Sources

This population-based propensity score-matched cohort study was performed using data from the Longitudinal Health Insurance Database 2000 (LHID2000), which contains the original claims data (collected between 1996 and 2011) of 1,000,000 individuals randomly sampled from the National Health Insurance (NHI) Research Database (NHIRD) in 2000 [28]. The NHIRD is an original database that contains the claims data of beneficiaries enrolled in the NHI program launched by the Taiwanese government in 1995. This single-payer, state-run program had covered 99.9% of Taiwan’s 23 million residents by 2004 and provides affordable, comprehensive, and compulsory health care services [29].

The description of the NHIRD is provided in our previous study [30]. This study was ethically approved by the Institutional Review Board of China Medical University and the Hospital Research Ethics Committee (Institutional Review Board approval number: CMU-REC-101-012). The requirement of informed consent from study participants was waived by the board committee because this study used secondary de-identified data released by the NHIRD.

### 2.2. Study Population

In this study, we identified 18,299 patients who had received a diagnosis of chronic HCV infection for the first time (International Classification of Diseases, Ninth Revision, Clinical Modification [ICD-9-CM] codes 070.41, 070.44, 070.51, 070.54, 070.70, 070.71, and V02.62) between January 1, 2000, and December 31, 2012. We excluded patients who had received only one diagnosis of acute or unspecified HCV infection (ICD-9-CM codes 070.41, 070.51, 070.70, and 070.71). Only patients who had received a second diagnosis of HCV infection within 6 months after being diagnosed as having acute or unspecified HCV infection were considered as having chronic HCV infection. The date of the first diagnosis of chronic HCV infection was selected as the index date.

**Figure 1:**
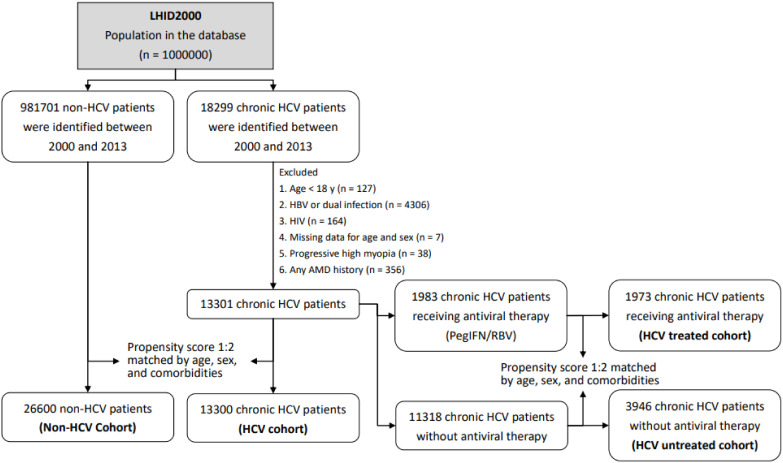
Study sample selection. LHID 2000: Longitudinal Health Insurance Database 2000.

The main objective of this study was to evaluate whether an association exists between AMD, including exudative and nonexudative types, and chronic HCV infection. We identified patients who had received a diagnosis of AMD (exudative type [ICD-9-CM codes 362.42, 362.43, 362.52, and 362.53] and nonexudative type [ICD-9-CM codes 362.50 and 362.51]) to examine the association between AMD and HCV infection. In addition, patients who were aged <18 years (n = 127); were diagnosed as having only HBV infection (ICD-9-CM codes 070.2, 070.3, and V02.61), dual infection of HBV and HCV (n = 4,306), or HIV infection (ICD-9-CM codes 042–044; n = 164); had missing data for age and sex (n = 7); and had a history of progressive high myopia (ICD-9-CM code 360.21; n = 38); or AMD diagnosis (n = 356) were excluded (Figure 1).

Finally, 13,300 patients diagnosed as having chronic HCV infection were included in the HCV cohort. To reduce potential confounding, we used propensity score matching at a 1:2 based on age, sex, and comorbidities for the HCV cohort. Propensity score matching criteria included age, sex, occupation, urbanization, monthly income, index year, myocardial infarction (MI), cerebrovascular disease (CVD), diabetes mellitus, renal disease, hypertension, hyperlipidemia, liver cirrhosis, anemia, and statin use. After matching, 26,600 patients without HCV infection were identified from 981,701 patients between 2000 and 2013 in the LHID2000 and were included in the non-HCV cohort (Figure 1).

From the HCV cohort, 1,983 patients who received antiviral pegylated interferon and ribavirin (PegIFN/RBV) treatment (Anatomical Therapeutic Classification codes L03AB04, L03AB05, L03AB09, L03AB10, L03AB11, L03AB60, and L03AB61) for ≥24 weeks were identified after matching their claims data to those in the NHI Strengthens Chronic Hepatitis B and C Treatment Plan. In this government-subsidized treatment plan, the levels of anti-HCV, HCV RNA, and liver enzymes are recorded before and after treatment periodically. A total of 1,973 of the 1,983 patients with HCV who completed PegIFN/RBV treatment (HCV treated cohort) and 3,946 chronic HCV carriers who did not receive antiviral therapy (HCV untreated cohort) were successfully matched using propensity scores at a ratio of 1:2 based on age; monthly income; index year; presence of MI, CVD, renal disease, cataract, liver cirrhosis, and anemia; and statin use (Figure 1).

### 2.3. Statistical Analysis

We first examined the distribution of sociodemographic factors, comorbidities, and medicine use between patients with and without chronic HCV infection through propensity score matching. The incidence rates of AMD and its subtypes (exudative and nonexudative) in the cohorts were calculated from the index date to the end of 2013. We calculated the person-years (PYs) of the follow-up duration for all participants until they received a diagnosis of AMD or until their enrollment was discontinued from the NHI, migration, or death, as determined in the NHIRD. Univariate and multivariate Cox proportional hazard regression models were used to calculate crude hazard ratios (cHRs) and adjusted hazard ratios (aHRs) and 95% confidence intervals (CIs) associated with the risk of AMD between the HCV and non-HCV cohorts under the Poisson assumption. HRs were adjusted for matched pairs and competing mortality in the multivariate Cox proportional hazard models. To examine whether the relationship between HCV infection and AMD development differed among different age groups and by sex, we performed subgroup analysis. Patients were classified into the following age groups: <50, 50–65, and ≥65 years. To assess whether aging and/or sex significantly interacts with HCV infection, resulting in AMD development, we performed Cox regression analysis and included a product term of age groups and/or sex and HCV infection status. In addition, we conducted sensitivity analysis to compare HRs before and after excluding cases identified within the first year after the index date. All models were examined with the log minus log survival plots by using the Schoenfeld’s test to ensure the adherence of the proportional hazards assumption. To assess the competing risk, the cumulative incidence of AMD in HCV carriers was calculated. Significant differences between the cohorts were evaluated using Gray’s test [31,32]. All analyses were performed using the Statistical Analysis Software package, version 9.4 (SAS Institute, Inc., Cary, NC, USA). A *p* value of <0.05 was considered statistically significant.

## 3. Results

### 3.1. Baseline Characteristics of Patients

Table 1 shows the comparisons between the HCV and non-HCV cohorts as well as between the HCV-treated and HCV-untreated cohorts after propensity score matching. All potential confounders were comparable between the HCV and non-HCV cohorts and between HCV-treated and HCV-untreated cohorts, except for cataract, which exhibited a slightly higher frequency in the HCV cohort than in the non-HCV cohort (*p* = 0.041).

### 3.2. Overall Risk of Any Type and Subtypes of AMD in Patients with Chronic HCV Infection

The median follow-up duration from the index date to the diagnosis of any type of AMD was 5.74 and 5.83, years, respectively, in the non-HCV and HCV cohorts, respectively. As shown in Table 2, the incidence of any type of AMD was 11.89 per 1000 PYs, which was equal to the combined incidence for both the non-HCV (5.32 per 1000 PYs) and HCV (6.57 per 1000 PYs) cohorts. In the multivariate Cox proportional hazards regression model, the aHR for any type of AMD in the HCV cohort was 1.22 (95% CI = 1.09–1.35). Furthermore, HCV infection was linked with a higher risk of nonexudative AMD (aHR = 1.22, 95% CI = 1.09–1.37); however, no association was observed between HCV infection and exudative AMD (aHR = 1.17, 95% CI = 0.84–1.61). In addition, compared with the HCV-untreated cohort, the HCV-treated cohort showed no significant association with any type of AMD (aHR = 1.07, 95% CI = 0.81–1.43) and its subtypes (exudative AMD: aHR = 1.36, 95% CI = 0.58–3.22 and nonexudative AMD: aHR = 1.04, 95% CI = 0.77–1.41).

**Figure 2:**
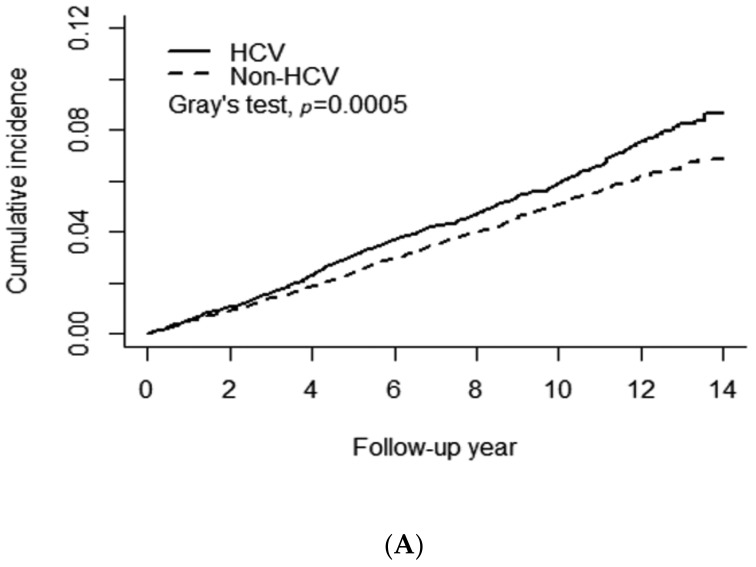

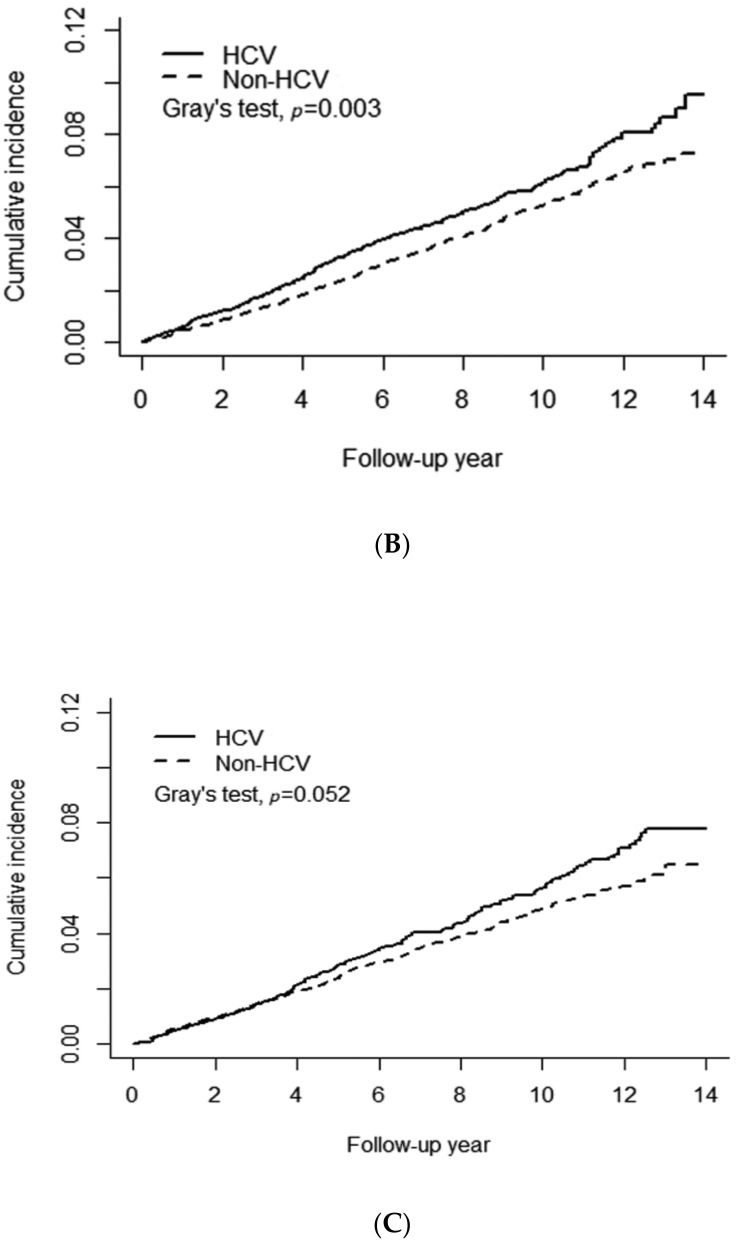
Association of chronic HCV infection with the risk of age-related macular degeneration. (**A**) All patients, (**B**) Female patients, (**C**) Male patients.

**Figure 3:**
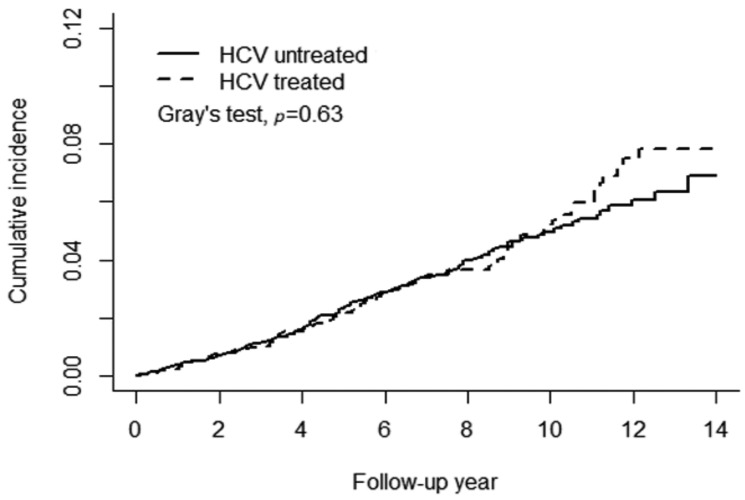
PegIFN/RBV therapy did not reduce the risk of age-related macular degeneration.

Figure 2A shows a significant difference in the cumulative risk of any type of AMD between the HCV and non-HCV cohorts (Gray’s test *p* = 0.0005). The 14-year cumulative incidence of any type of AMD was approximately 0.087 and 0.069 in the HCV and non-HCV cohorts, respectively. The observed difference between the HCV and non-HCV cohorts remained significant after stratification for female patients with Gray’s test *p* = 0.003 (Figure 2B) but not for males (*p* = 0.052) (Figure 2C). Figure 3 presents the results of the analysis of the administration of anti-viral PegIFN/RBV therapy in patients with HCV. No association between the therapy and the risk of AMD was observed in patients with HCV. The cumulative incidence of AMD in the HCV-treated and HCV-untreated cohorts was 0.078 and 0.069, respectively (Gray’s test *p* = 0.63).

### 3.3. Age and Sex-Specific Risks of AMD in Relation to Chronic HCV Infection

As shown in Table 3, the results of age-specific analysis revealed that patients with chronic HCV infection aged <50 years had the highest likelihood of having AMD (aHR = 1.46, 95% CI = 1.03–2.07, *p* = 0.03), followed by those aged ≥65 years (aHR = 1.19, 95% CI = 1.03–1.38, *p* = 0.02) and those aged between 50 and 64 years (aHR = 1.17, 95% CI = 0.98–1.41, *p* = 0.09). The results of sex-specific analysis indicated that the risk of AMD in patients with HCV infection was similar between men (aHR = 1.17, 95% CI = 1.01–1.37) and women (aHR = 1.25, 95% CI = 1.08–1.45). As shown in Table 3, no significant association between any type of AMD in HCV carriers and PegIFN/RBV treatment status was observed, even after stratification by either age or sex. These findings indicated that age and sex did not interact with HCV infection or treatment for the risk of any type of AMD in this study population (all *p* values > 0.05; Table 3). 

### 3.4. Risk of AMD in Patients with Chronic HCV Infection after Excluding Cases Diagnosed within the First Year after the Index Date

The results of multivariate Cox proportional hazards regression analysis (Table 4) revealed a notable risk (aHR = 1.25, 95% CI = 1.11–1.40, *p* = 0.0002) of any type of AMD among the HCV and non-HCV cohorts after excluding cases diagnosed within the first year after the index date. The HRs calculated from the models before and after excluding cases diagnosed in the first year did not change risk estimates by more than 2.5%. No significant risk of any type of AMD was observed in patients with chronic HCV infection receiving PegIFN/RBV treatment (aHR = 1.10, 95% CI = 0.82–1.48, *p* = 0.520).

## 4. Discussion

The results of this population-based retrospective cohort study revealed a positive association of any type of AMD and nonexudative AMD with chronic HCV infection. This positive association was unlikely to be attributed to antiviral PegIFN/RBV treatment but rather was likely to be related to HCV infection itself. Although not significant, the observed association between AMD and HCV infection was found to be stronger in patients aged <50 years. In addition, to prevent the potential confounding effect of the co-existence of AMD and HCV infection, we excluded AMD cases diagnosed within the first year after the HCV index date in subgroup analysis and found a stronger positive association between AMD risk and HCV infection. To the best of our knowledge, this is the first study to provide population-based evidence for a new risk factor for AMD apart from the already established risk factors such as age.

AMD is categorized into nonexudative (non-neovascular) and exudative (neovascular) types. The major pathological presentations of nonexudative AMD are drusen formation, retinal pigmentary epithelium (RPE) abnormalities, geographic atrophy, and hyperpigmentation. Exudative AMD initiates choroidal neovascularization under the retina and consequently causes a centrally blinding disciform scar if left untreated [33]. In a recent case study, Quillen et al. [22] reported the case of a 22-year-old woman who developed acute retinal pigment epithelitis in the right eye during her acute HCV infection stage [22]. This finding suggests that HCV can directly infect the retina and cause RPE abnormalities. Damage to the RPE can lead to the accumulation of metabolic waste products and subsequent damage to Bruch’s membrane or choroidal capillaries [34].

The specific mechanism through which chronic HCV infection causes AMD remains unclear. The pathogenesis of ocular changes may be the direct action of viral and immunological reactions to certain viral antigens and even immune complexes [27]. On the basis of the findings of their in vitro experiments, Pazienza [35] reported that HCV itself possesses regulatory genes that can cause ocular pathology. HCV core expression can deregulate the mRNA levels of (a) uveal autoantigen with coiled-coil domains and ankyrin repeats, (b) polymerase DNA-directed gamma, (c) RB-associated KRAB repressor, (d) Ras-related protein, and (e) retinoblastoma [35]. Alternatively, HCV itself may cause abnormalities in complement production and regulation, leading to a decline in the complements C3 and C4 and the complement factor H-related protein 1. These mechanisms can lead to the activation of alternative complement pathways and inflammation processes. The whole cascade may increase the formation of drusen and may increase the risk of AMD [36,37].

This is the first study to report an association between AMD and HCV. This finding may be attributed to the following factors. In Western countries, the use of drug abuse-related intravenous injection is a major route of HCV transmission, predominantly during young adulthood [38]. By contrast, in Taiwan, in earlier decades, the inapt cultural belief in intravenous injections for minor conditions such as fatigue and common cold, the reuse of syringes without adequate sterilization, and the illegal medical practices of unlicensed health providers were the common causes of HCV infection [11,39]. Thus, compared with people in Western countries, people in Taiwan have been exposed to the risk of HCV infection for a longer time. The prolonged increased risk of HCV infection in Taiwan could have promoted AMD development by enhancing the inflammatory process in target ocular tissues.

In addition, the variation in HCV genotyping may partially suggest a positive association between AMD and HCV infection in our study. HCV type 1a and 1b are predominantly observed in the population in the United States and Europe [40]. However, according to a study including 1164 Taiwanese patients with positive HCV antibodies and HCV RNA (ribonucleic acid), genotype 1b is more frequently detected in the older population, whereas genotype 2a is more common in younger adults [41]. In 2019, a study revealed that 18.3% of serum samples obtained from 1147 hepatitis C carriers in southern Taiwan exhibited genotype 6 [42]. Therefore, HCV genotypes 1a and 1b, which are prevalent in the United States and Europe, are unlikely to be associated with AMD in Taiwan. Further studies are needed to apply these findings to other regions.

Many ocular manifestations are suggested to be associated with the adverse treatment effects of PegIFN/RBV [25]. In our subgroup analysis, we observed that the risk of AMD (aHR = 1.07, 95% CI = 0.81–1.43) did not significantly differ between the HCV treated and HCV untreated cohorts. This finding suggests that the association between AMD and HCV infection is most likely due to HCV infection itself. The use of ICD-9-CM codes to classify diseases may create coding variants. However, our HCV treated cohort was identified by matching the data of the NHI Strengthens Chronic Hepatitis B and C Treatment Plan; according to this plan, treating hepatologists are required to periodically submit patients’ anti-HCV, HCV RNA, and HCV genotype results and related biochemical and imaging study findings to the NHI for antiviral treatment [43]. Earlier studies have suggested that more than 70% of Taiwanese patients with positive anti-HCV antibodies also tested positive for HCV RNA [44,45,46]. Moreover, <10% of 400,000 Taiwanese HCV carriers with positive RNA successfully achieved a sustained viral response after the completion of interferon therapy prior to 2011 [47]. Furthermore, the diagnosis of AMD is primarily and specifically established and coded by ophthalmologists. Therefore, we believe that ICD-9-CM codes can provide a relatively representative prevalence of HCV infection in Taiwan.

This study has several limitations that should be addressed. First, asymptomatic HCV carriers may not seek any medical attention and thus would not be identified based on ICD-9-CM codes in this study. Therefore, these patients might have been incorrectly included in the non-HCV cohort. This mis-categorization may have led to estimated HRs toward null values, thus weakening the estimation of the association. Second, with the use of ICD-9 CM codes from the NHIRD, the interaction between the severity of HCV infection and AMD could not be further analyzed because related liver function and HCV RNA data could not be obtained. Furthermore, family history and genetic data of AMD are not included in the NHIRD; thus, we could not assess the interference of genetic factors. Fourth, smoking is a well-known risk factor for AMD; however, patients’ smoking status is not provided in the NHIRD. Chronic obstructive pulmonary disease (COPD) is linked with cigarette smoking. Although we adjusted for COPD instead of smoking, some potential confounding effects related to smoking risk could not be eliminated. And finally, other ethnic groups may not share similar HCV transmission routes as those in Taiwan. Hence, the generalization of the results of this study should be conducted cautiously.

## 5. Conclusions

The results of this population-based retrospective cohort study suggest an increased risk of AMD in Taiwanese patients with chronic HCV infection. Additional large-scale epidemiological studies should be performed among different ethnic populations to determine the underlying pathophysiological association between AMD and HCV infection.

## Figures and Tables

**Table 1:** Sociodemographic characteristics and comorbidities of the HCV and non-HCV cohorts with propensity score matching.

Parameters	Non-HCV (n = 26,600)	HCV (n = 13,300)	*p* Value	HCV-Untreated (n = 3,946)	HCV-Treated (n = 1,973)	*p* Value
Age, year	55.6 (17.0)	55.5 (15.6)	0.464	51.1 (14.3)	51.0 (11.6)	0.686
Male sex, N (%)	13,395 (50.4)	6662 (50.1)	0.253	2101 (53.2)	1078 (54.6)	0.311
Occupation, N (%)			0.720			0.290
White collar	10,896 (41.0)	5430 (40.8)		1621 (41.1)	852 (43.2)	
Blue collar	13,353 (50.2)	6723 (50.6)		2059 (52.2)	989 (50.1)	
Others	2351 (8.84)	1147 (8.62)		226 (6.74)	132 (6.69)	
Urbanization, N (%)			0.466			0.170
Urban	5727 (21.5)	2887 (21.7)		854 (21.6)	435 (22.1)	
Suburban	1196 (45.0)	5933 (44.6)		1798 (45.6)	938 (47.5)	
Rural	8912 (33.5)	4480 (33.7)		1294 (32.8)	600 (30.4)	
Geographical region, N(%)			0.154			0.697
Northern	7610 (28.6)	3923 (29.5)		1168 (29.6)	565 (28.6)	
Central	6091 (22.9)	2937 (22.1)		855 (21.7)	415 (21.0)	
Southern	11,375 (42.8)	5694 (42.8)		1719 (43.6)	884 (44.8)	
Eastern and islands	1524 (5.73)	746 (5.61)		204 (5.17)	109 (5.52)	
Monthly Income (NT$), N(%)			0.977			0.652
<15,840	8699 (32.7)	4361 (32.8)		1038 (26.3)	518 (26.3)	
15,840–24,999	13,360 (50.2)	6665 (50.1)		2058 (52.2)	1010 (51.2)	
≥25,000	4541 (17.1)	2274 (17.1)		850 (21.5)	445 (22.6)	
Comorbidities, N (%)						
MI	6144 (23.1)	357 (23.0)	0.801	556 (14.1)	282 (14.3)	0.833
CVA	4003 (15.1)	1988 (15.0)	0.789	303 (7.68)	167 (8.46)	0.292
COPD	9921 (37.3)	5066 (38.1)	0.123	1283 (32.5)	645 (32.7)	0.891
DM	6793 (25.5)	3352 (25.2)	0.469	856 (21.7)	428 (21.7)	1.000
Renal disease	2148 (8.08)	1121 (8.43)	0.225	127 (3.22)	75 (3.80)	0.244
HTN	11,760 (44.2)	5878 (44.2)	0.977	1396 (35.4)	687 (34.8)	0.672
Hyperlipidemia	2962 (11.1)	1420 (10.7)	0.167	390 (9.88)	189 (9.58)	0.710
Cataract	4426 (16.6)	2321 (17.5)	0.041	392 (9.93)	203 (10.3)	0.669
Diabetic retinopathy	526 (1.98)	288 (2.17)	0.211	46 (1.17)	32 (1.62)	0.147
Liver cirrhosis	18,626 (70.0)	9318 (70.1)	0.938	3287 (83.3)	1642 (83.2)	0.941
Obesity	464 (1.74)	223 (1.68)	0.624	75 (1.90)	34 (1.72)	0.632
Anemia	4169 (15.7)	2152 (16.2)	0.191	430 (10.9)	212 (10.8)	0.859
Medication, N (%)						
Statin	1055 (3.97)	545 (4.10)	0.528	91 (2.31)	48 (2.43)	0.762
Aspirin	34 (0.13)	19 (0.14)	0.697	8 (0.20)	1 (0.05)	0.289
NSAIDs	127 (0.48)	71 (0.53)	0.450	16 (0.41)	8 (0.41)	1.00

Data are expressed as the mean (standard deviation) or number (percentage). CVA: Cerebrovascular disease; COPD: chronic pulmonary disease; DM: diabetes mellitus; HCV: hepatitis C virus; HTN: hypertension; MI: myocardial infarction; NSAIDs: Nonsteroidal anti-inflammatory drugs.

**Table 2:** Multivariate Cox proportional hazards regression analysis for the risk of AMD in patients with chronic HCV.

AMD		Non-HCV	HCV	*p* Value		HCV-Untreated	HCV-Treated	*p* Value
Any type	Cases	852	535		Cases	127	75	
	Person-year	160,053	81,460		Person-year	24,710	13,912	
	Incidence (10^−3^)	5.32	6.57		Incidence (10^−3^)	5.14	5.39	
	HR ^a^ (95% CI)	1.00 (ref)	1.22 (1.09–1.35)	0.0004	HR ^a^ (95% CI)	1.00 (ref)	1.07 (0.81–1.43)	0.240
Exudative	Cases	98	59		Cases	12	9	
	Person-year	160,053	81460		Person-year	24,710	13,912	
	Incidence (10^−3^)	0.61	0.72		Incidence (10^−3^)	0.49	0.65	
	HR ^a^ (95% CI)	1.00 (ref)	1.17 (0.84–1.61)	0.354	HR ^a^ (95% CI)	1.00 (ref)	1.36 (0.58–3.22)	0.497
Nonexudative	Cases	754	476		Cases	115	66	
	Person-year	160,053	81,460		Person-year	24,710	13,912	
	Incidence (10^−3^)	4.71	5.84		Incidence (10^−3^)	4.65	4.74	
	HR ^a^ (95% CI)	1.00 (ref)	1.22 (1.09–1.37)	0.0006	HR ^a^ (95% CI)	1.00 (ref)	1.04 (0.77–1.41)	0.781

AMD: age-related macular degeneration; CI: confidence interval; HCV: hepatitis C virus; HR: hazard ratio; ref: reference. ^a^ Adjusted for matched pairs and competing mortality.

**Table 3:** Age- and sex-specific multivariate Cox proportional hazards regression analysis for the risk of AMD according to HCV infection and treatment status.

	Age (years)						Sex			
	<50		50–64		≥65		Males		Females	
Parameter	HR ^a^ (95% CI)	*p*	HR^a^ (95% CI)	*p*	HR ^a^ (95% CI)	*p*	HR ^a^ (95% CI)	*p*	HR ^a^ (95% CI)	*p*
Non-HCV	1.00 (ref)		1.00 (ref)		1.00 (ref)		1.00 (ref)		1.00 (ref)	
HCV	1.46 (1.03–2.07)	0.03	1.17 (0.98–1.41)	0.09	1.19 (1.03–1.38)	0.02	1.17 (1.01–1.37)	0.043	1.25 (1.08–1.45)	0.003
Interaction		0.46						0.53		
HCV-untreated	1.00 (ref)		1.00 (ref)		1.00 (ref)		1.00 (ref)		1.00 (ref)	
HCV-treated	1.12 (0.56–2.22)	0.75	0.96 (0.64–1.44)	0.84	1.45 (0.89–2.38)	0.14	0.87 (0.56–1.33)	0.507	1.28 (0.88–1.87)	0.198
Interaction		*p* = 0.37						0.19		

AMD: age-related macular degeneration; CI: confidence interval; HCV: hepatitis C virus; HR: hazard ratio; ref: reference. ^a^ Adjusted for matched pairs and competing mortality.

**Table 4:** Multivariate Cox proportional hazards regression analysis for the risk of AMD after excluding cases diagnosed as having AMD within the first year after the index date.

Parameters	HR ^a^ (95% CI)	*p* Value
Non-HCV	1.00 (Reference)	
HCV	1.25 (1.11–1.40)	0.0002
HCV untreated	1.00 (Reference)	
HCV treated	1.10 (0.82–1.48)	0.520

AMD: age-related macular degeneration; CI: confidence interval; HCV: hepatitis C virus; HR: hazard ratio. ^a^ Adjusted for matched pairs and competing mortality.

## Data Availability

Data were first retrieved from the NHIRD of Taiwan (http://nhird.nhri.org.tw/) on 01 October 2015, and access to this database can be requested by sending a formal proposal to the NHI.

## Abbreviations

AMD	age-related macular degeneration
COPD	chronic pulmonary disease
CVD	cerebrovascular disease
CI	confidence interval
HBV	hepatitis B virus
HCV	hepatitis C virus
HIV	human immunodeficiency virus
HR	hazard ratio
aHR	adjusted hazard ratio
ICD-9-CM	International Classification of Diseases, Ninth Revision, Clinical Modification
NHI	National Health Insurance
NHIRD	National Health Insurance Research Database
MI	myocardial infarction
PegIFN/RBV	anti-viral pegylated interferon and ribavirin treatment
PYs	person years
RNA	ribonucleic acid
